# Protective Effect of Crocin on Gastric Mucosal Lesions Induced by Ischemia-Reperfusion Injury in Rats

**Published:** 2016

**Authors:** Seyyed Ali Mard, Seyyed Mojib Azad, Akram Ahangarpoor

**Affiliations:** a *Physiology Research Center (PRC), Research institute for infectious diseases of digestive system and Department of Physiology, School of Medicine, Ahvaz Jundishapur University of Medical Sciences, Ahvaz, Iran. *; b *Physiology Research Center (PRC), and Department of Physiology, School of Medicine, Ahvaz Jundishapur University of Medical Sciences, Ahvaz, Iran.*

**Keywords:** Crocin, Superoxide dismutase, Glutathione peroxidase, Ischemia-reperfusion injury, Rat

## Abstract

The present study aimed to evaluate the protective effect of crocin on gastric mucosal lesions caused by ischemia-reperfusion (I/R) injury in rats. Forty male rats were randomly divided into sham, control (I/R injury) and three crocin-pretreated groups. To induce I/R lesions, the celiac artery was clamped for 30 min and then the clamp was removed to allow reperfusion for 3 h. Pretreated-rats received crocin (7.5, 15 or 30 mg/kg, i.p.) 30 min prior to the induction of I/R injury. Samples of gastric mucosa were collected to measure the following variables: 1 mRNA expression of superoxide dismutase (SOD) and glutathione peroxidase (Gpx) by RT-PCR; 2 activity of superoxide dismutase and glutathione peroxidase and 3 tissue levels of malonyldehaldehyde (MDA).

Pretreatment with crocin decreased the total area of gastric lesions. Messenger RNA expressions of SOD and Gpx in control I/R injury rats were significantly decreased as compared with sham-operated group (P<0.001). Crocin pretreatment 30 min prior to I/R injury significantly increased mRNA expressions of SOD and Gpx genes. The gastric mucosal activities of SOD and Gpx in control I/R injury rats were significantly lower than in crocin-pretreated groups (P<0.01). Crocin pretreatment decreased mucosal production of MDA.

Our findings showed the protective effect of crocin on gastric mucosa against ischemia-reperfusion injury. These effects of crocin were mainly mediated by increasing the mRNA expressions- and the enzyme activity of SOD and Gpx as well as by inhibiting the production of free radicals.

## Introduction

It has been shown that reactive oxygen species (ROS) such as superoxide, hydrogen peroxide, and hydroxyl radicals play a major role (are involved) in the development of ischemia-reperfusion injury in a variety of tissues, including the stomach (1, 2). Under normal conditions, the endogenous antioxidant enzymes such as SOD and GSH neutralize reactive radicals of oxygen molecules produced during metabolism and prevent their destructive effects (3). However, when there was an imbalance between the production of oxidants and the antioxidant defense systems, the oxidative stress occurs. The human body possesses many natural antioxidants; however, none of them are capable of protection against the attack of oxidants induced by the ischemia-reperfusion condition (4). Antioxidant agents have demonstrated to protect the gastrointestinal mucosa against I/R-induced damages in many animal reports (5-7). 

Crocin, the most important and abundant antioxidant constituent of *Crocus sativus* stigma (8), has been demonstrated to exert antioxidant property (9). Its beneficial effects has been shown in different models of experimentally induced gastric ulcer models such as NSAIDs (10)-, pylorus ligated (11)-, and water immersion restraint stress (12). To our knowledge, no previous study has specifically investigated the possible protective effect of crocin on gastric mucosal following I/R injury in rat. Therefore, the aim of the present study was to evaluate the protective effect of crocin on gastric mucosal lesions induced by I/R injury in rat by evaluating the changes in the level of mRNA expression and enzyme activity of superoxide dismutase and glutathione peroxidase.

## Experimental

*Chemicals and Animals*

Crocin (Cat NO-17304) was purchased from Sigma (USA). Male Wistar rats (body weight 130-160g) were purchased from the animal house of Ahvaz Jundishapur University of Medical Sciences. The animals were fed on conventional diets and had free access to tap water. They were maintained under standard conditions of humidity, temperature (22 ± 2 ºC) and light/dark cycle (12 h:12 h). The animals were deprived of food but not water 16 h before the experiment. All experiments were carried out in accordance with ethics committee of Ahvaz Jundishapur University of Medical Sciences (PRC144).

*Animal grouping and surgical procedures*

Forty male Wistar rats were randomly assigned to one of 5 groups (n=8): Sham, control (gastric ischemia-reperfusion; I/R injury) and 3 crocin-pretreated groups. Gastric I/R injury was induced according to the method of Wada (13). Briefly, under a sodium pentobarbital anesthesia (50 mg/kg, i.p.), the rats underwent a midline laparatomy and the celiac artery was carefully isolated from its adjacent tissues. The celiac artery was then clamped by a ligature for 30 min to induce ischemia and the ligature was removed to allow reperfusion for 3 h. Sham-operated rats underwent laparatomy without inducing I/R injury. To investigate the gastroprotective effect of crocin against mucosal damage induced by I/R injury, 3 groups of animals received crocin (i.p.) at doses of 7.5, 15 or 30 mg/kg 30 min prior to I/R injury. At the end of experiment, animals were killed by cardiac exsanguination. In order to calculate the gastric mucosal lesions, the stomachs of animals were removed, opened along the greater curvature, rinsed with physiological saline and pinned out in ice-cold saline. To calculate the degree of gastric lesions, the total area of mucosal lesions were measured by Image J software. The lesion area is expressed as a percentage of the total area of the glandular stomach except for the fundus using following formula : UI(%)=[Ulcerated area/total stomach area expect fundus]×100 (14). Immediately after taking photo of the stomachs for measurement of the surface area of gastric lesions, two samples of gastric mucosal tissue (50 mg in each) including the lesions area and the surrounding ulcer margin were quickly excised, snap-frozen and stored in liquid nitrogen for molecular analysis, determination of enzyme activity and lipid peroxidation. The macroscopic evaluation of gastric mucosal lesions showed that the optimal protective dose of crocin against I/R injury was 15 mg/kg. Therefore, the molecular analysis was carried out in animals that received the optimal dose. 

*RNA extraction and cDNA synthesis*

The total RNA was extracted from the frozen tissue samples using RNeasy mini plus kit (Qiagen, USA). The concentration and purity of the total RNA was determined spectrophotometerically at 260 and 280 nm wavelength (Eppendorf, BioPhotometer *Plus*, Germany). The cDNA was synthesized from one microgram of the total RNA by using Quantitect Reverse Transcription kit (Qiagen, USA) according to the manufacturer´s instruction. 

*Reverse transcriptase PCR*

All PCR amplifications were performed in final volume of 25 µL containing 1µg cDNA, 50 nm of specific primers, 2.5 µL of 10X PCR buffer, 1 uDNA Taq polymerase and 50 nm of dNTP. The mRNA levels of SOD, Gpx and the housekeeping gene glyceraldehyde-3-phosphate dehydrogenase (GAPDH) were measured by RT-PCR using *Master Cycle Personnel* (Eppendorf AG, Hamburg, Germany). The specific primers (Bioneer, Daejoun, South Korea) used in this study are listed in Table1. The thermal cycling conditions for the amplification of GAPDH, SOD and Gpx genes were as follows: initial denaturation at 94 °C for 5 min followed by 40 cycles of 1 min at 94 °C; annealing time 60 s and the temperature was at 53 °C for GAPDH, 54 °C for SOD and 55 °C for Gpx and the elongation time was 1 min at 72 °C. A. final elongation cycle at 72 °C for 5 min was also performed. The PCR products were analyzed on a 2% agarose gel and the density of each band was measured with Image J software. The levels of the target studied genes; SOD and Gpx; were determined by calculating the density ratio of each studied mRNA/GAPDH mRNA. 

**Table 1:** Specific primers for GAPDH, SOD and Gpx

Primer sequence
SOD
F: 5´-GCAGAAGGCAAGCGGTGAAC-3´
R: 5´-TAGCAGGACAGCAGATGAGT-3´
Gpx
F: 5´-CTCTCCGCGGTGGCACAGT-3´
R: 5´-CCACCACCGGGTCGGACATAC-3´
GAPDH
F: 5´-TGC-TGG-TGC-TGA-GTA-TGT-CGT-G-3´
R: 5´-CGG-AGA-TGA-TGA-CCC-TTT-TGG-3´

*Measurement of* *superoxide dismutase and glutathione peroxidase* *activity.*

The activity of superoxide dismutase (SOD) and glutathione peroxidase (GPx) in the homogenates of gastric mucosal tissue were measured using a commercial kit (Biocore Diagnostik Ulm GmbH, Veltlinerweg 29, Deutschland) according to the manufacturer’s instructions.

***Determination of lipid peroxidation***

MDA levels were measured to show the level of lipid peroxidation. Briefly, in this method MDA reacts with thiobarbituric acid (TBA) as a thiobarbituric acid reactive substance (TBARS) to generate a red-colored complex that has peak absorbance at 532 nm. Three mL phosphoric acid (1 %) and 1 mL TBA (0.6 %) was added to 0.5 mL of homogenate and the mixture was heated for 60 min in a boiling water bath. Then, twenty-five µL HCl was added to the ice-cooled mixture and vortexed. At the end, 3.5 mL of n-butanol was added the mixture and incubated for 5 min and centrifuged at 15000 rpm for 10 min to separate n-butanol phase. The supernatant was transferred to a new tube and its absorbance was measured at 532 nm. The standard curve of MDA was constructed over the concentration range of 0-40 μM (9).

*Statistical analysis*

Data are shown as mean ± S.E.M. Statistical analysis was performed by one-way ANOVA and followed by post hoc Tukey’s test. Significance was set at a P<0.05 level.

## Results

*Effect of crocin pretreatment on gastric mucosal lesions induced by I/R injury*

**Figure 1:**
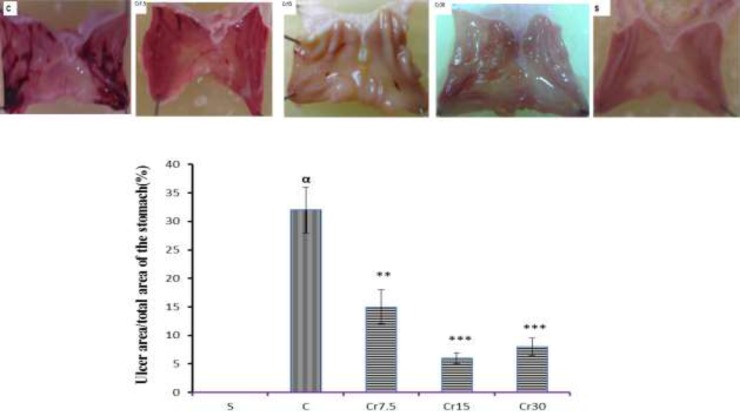
The macroscopic representation of gastric mucosal lesions. S: Sham-operated group show normal gastric mucosal tissue; C: control (I/R) group indicate gastric mucosal lesions; Cr: Pretreated groups (the animals received a single administration of crocin at 7.5, 15 or 30 mg/kg, 30 min before to I/R induction) demonstrate a significant reduction of gastric mucosal lesions

**Figure 2:**
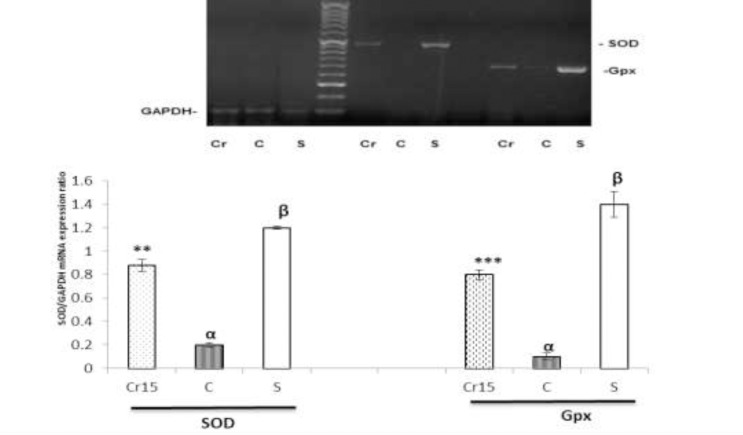
A graghic represenation of the ulcer index following ischemia-reperfusion injury among various treatment groups: S: sham, C: control and Cr: crocin-pretreated groups (the animals received a single administration of crocin at 7.5, 15 or 30 mg/kg, 30 min before to I/R induction). Crocin produced singificant dose-dependent reduction in ulcer index. ***P<0.001, **P<0.01 *versus* the control group and ^α^P<0.001 *versus* the Sham group. Data are expressed as mean±S.E.M

As shown in Figure 1, no macroscopic lesion was observed in the gastric mucosa of the normal rats in the sham-operated group. Pretreatment with crocin attenuated the gastric lesions induced by I/R injury (Figure 1). As shown in Figure 2, the total area of lesions induced by I/R injury was significantly decreased by pretreatment of crocin in a dose-dependent manner (P<0.01). The results also showed crocin at 15 mg/kg was the optimal protective dose. 

*Effect of crocin pretreatment on mucosal mRNA expressions of* *SOD and Gpx*

**Figure 3:**
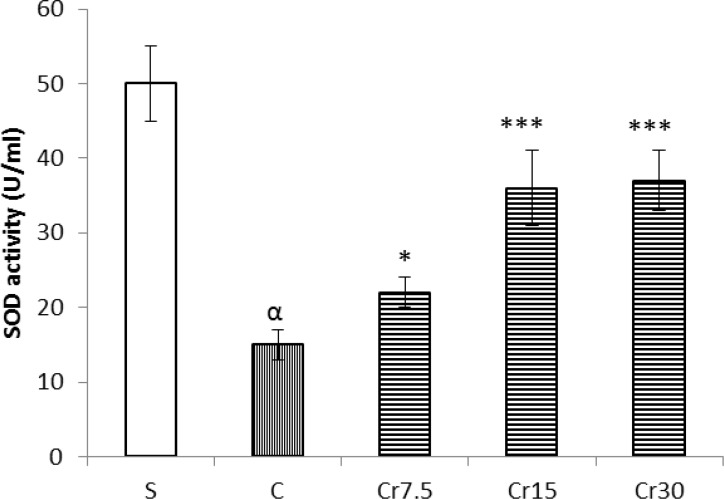
A graghic and agarose gel representive bands (for target and housekeeping genes) of the effect of crocin pretreatment (the animals received a single administration of crocin at 7.5, 15 or 30 mg/kg, 30 min before to I/R induction) on the gastric mucosal mRNA expression of superoxide peroxide and glutathione peroxidase. Analysis of RT-PCR results showed that the pre-administration of crocin increases the mRNA expression of SOD and Gpx. S: sham, C: control, Cr: crocin (15 mg/kg, i.p.); ^β^P<0.01 and ***P<0.001 *versus* the control group and ^α^P<0.001 *versus* the Sham group. Data are expressed as mean±S.E.M

Thirty min gastric ischemia followed by 3 h reperfusion significantly down regulated the basal level of messenger RNA expression of SOD and Gpx as compared with the sham-operated animals (P<0.001). As shown in Figure 3, crocin pretreatment was significantly reversed these reduction (P<0.01) (Figure 3).

*Effect of crocin pretreatment on* *SOD and Gpx activity*

**Figure 4:**
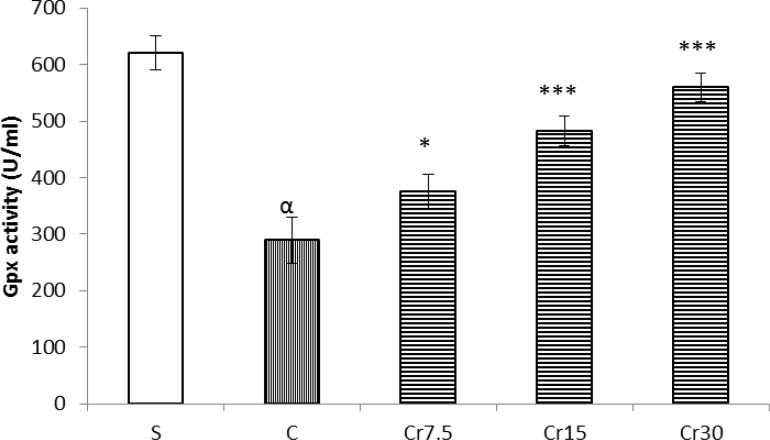
Effect of crocin pretreatment on SOD and Gpx activities in mucosal tissue of the stomach against I/R injury in rat. S: sham, C: control and Cr: crocin-pretreated groups (the animals received a single administration of crocin at 7.5, 15 or 30 mg/kg, 30 min before to I/R induction). Crocin were significantly reversed the activity of SOD and Gpx. ***P<0.001, *P<0.01 *versus* the control group and ^α^P<0.001 *versus* the Sham group. Data are expressed as mean ± S.E.M

As shown in Figure 4 (A and B), the activities of SOD and Gpx in mucosal tissue of the stomach in control I/R injury rats were significantly lower than in sham-operated animals (P<0.01). These levels were significantly increase by a single administration of crocin (P<0.01). 

*Effect of crocin pretreatment on lipid peroxidation in gastric mucosal tissue*

**Figure 5:**
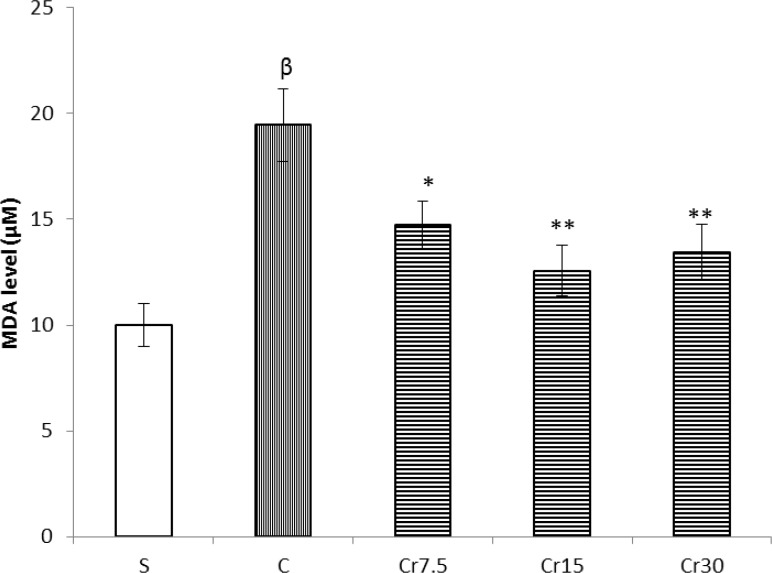
Effect of crocin pretreatment on MDA level in mucosal tissue of the stomach against I/R injury in rat. S: sham, C: control and Cr: crocin-pretreated groups (the animals received a single administration of crocin at 7.5, 15 or 30 mg/kg, 30 min before to I/R induction). Crocin pretreatment were significantly decreased the tissue levels of MDA. *P<0.05, **P<0.01 *versus* the control group and ^β^P<0.001 *versus* the Sham group. Data are expressed as mean ± S.E.M

Following gastric I/R injury, the mucosal level of MDA was significantly increased as compared with sham-operated rats (P<0.01). Free radical-induced lipid peroxidation was significantly decreased as indicated by a reduction in the MDA levels of gastric mucosal tissue by crocin pretreatment at three studied dose (7.5, 15 and 30 mg/kg) (P<0.05, Figure 5). 

## Discussion

 The results of the present study showed that a single administration of crocin protected the gastric mucosa against ischemia-reperfusion injury in rats. It has been shown that SOD attenuated the total area of gastric lesions in rats (15). Aqueous extract of saffron exhibited significant antiulcer activity in rat. The mechanism of this protection was proposed to be mediated by decrease in gastric non-protein sulfhydryl contents (16). In addition crocin and safranal produced their gastroprotective effects against indomethacin, in both diabetic and non-diabetic rats, by increasing gluthatione level and diminishing the lipid peroxidation. Moreover, it has been reported the neuroprotective mechanism of crocin against cerebral ischemia is mediated through suppression of free radical production by inhibiting lipid peroxidation (16).Consistent with these results; the present study revealed that crocin pretreatment decreased the tissue levels of MDA (Figure 5). Therefore, crocin protected the gastric mucosal tissue against I/R injury through reducing the production of free radicals. 

Vakili and Hosseinzadeh in their reports have been shown that the neuro-and reno-protective effects of crocin against ischemia-reperfusion injury is mediated through an increase the activity of SOD and Gpx (9, 16). The results of this study are also in agreement with these findings that showed crocin pretreatment increased the activity of SOD and Gpx in gastric mucosa. Therefore, these reports together show that protective effect of crocin on I/R injury could largely mediated through an increase the activity of antioxidant. 

It has been shown that mRNA expression of antioxidants, SOD and Gpx, down-regulated following oxidative stress (17). Consistent with these results, our results showed that the gene expression of SOD and Gpx significantly decreased after gastric I/R injury. Therefore, following ischemia-reperfusion injury, there is an increase the production of free radicals due to a reduction in antioxidants which it in turn damage tissues. The present study revealed that crocin pretreatment increased the mRNA expressions of superoxide dismutase and glutathione peroxidase in mucosal tissue of the stomach in male Wistar rat. Therefore, the other possible gastroprotective mechanism of crocin could largely be mediated by up-regulating the antioxidants, SOD and Gpx. 

Hosseinzadeh and his colleagues have been shown that the reno-protective effect of crocin on renal ischemia/reperfusion injury significantly increased as a dose dependent manner (9). They showed that crocin protected the renal function by inhibiting lipid peroxidation. The antioxidant potency of crocin at 200 and 400 mg/kg was identical as if its inhibitory effect on lipid peroxidation and the level of MDA concentration between two group that pretreated by 200 and 400 mg/kg crocin was similar (9). Similarly this study showed that crocin at 15 and 30 mg/kg had the same protective effect against I/R injury. 

As shown in results the total area of mucosal lesions in group 3 that received 7.5 mg/kg of crocin was higher than in group 4 (15 mg/kg of crocin) (Figure 2). However, the findings also indicated that the protective role of the highest studied dose of crocin (30 mg/kg) was similar to 15 mg/kg of crocin. Therefore, the optimal protective dose of crocin on gastric ischemia/reperfusion injury was 15 mg/kg. Taken together, these results suggest that the protective effect of crocin at lower doses (15 mg/kg) may increase the safety of crocin on gastric I/R injury (as shown by the present study) and on renal I/R injury (as shown by previous study) (9). 

In conclusion, the present study, for the first time, showed that the gastroprotective effect of crocin on I.R injury. The findings of this study demonstrated that: Pretreatment with crocin decreased the total area of acute gastric mucosal lesions induced by I/R in a dose-dependent manner. Crocin pretreatment was upregulated mRNA expressions of SOD and Gpx. The activity of SOD and Gpx were increased by crocin pretreatment. Crocin pretreatment was reduced lipid peroxidation. 
